# Investigating accountability for Artificial Intelligence through risk governance: A workshop-based exploratory study

**DOI:** 10.3389/fpsyg.2023.1073686

**Published:** 2023-01-25

**Authors:** Ellen Hohma, Auxane Boch, Rainer Trauth, Christoph Lütge

**Affiliations:** ^1^School of Social Sciences and Technology, Institute for Ethics in AI, Technical University of Munich, Munich, Germany; ^2^School of Engineering and Design, Chair of Automotive Technology, Technical University of Munich, Munich, Germany

**Keywords:** accountability, Artificial Intelligence, organizational framework, risk governance, workshop

## Abstract

**Introduction:**

With the growing prevalence of AI-based systems and the development of specific regulations and standardizations in response, accountability for consequences resulting from the development or use of these technologies becomes increasingly important. However, concrete strategies and approaches of solving related challenges seem to not have been suitably developed for or communicated with AI practitioners.

**Methods:**

Studying how risk governance methods can be (re)used to administer AI accountability, we aim at contributing to closing this gap. We chose an exploratory workshop-based methodology to investigate current challenges for accountability and risk management approaches raised by AI practitioners from academia and industry.

**Results and Discussion:**

Our interactive study design revealed various insights on which aspects do or do not work for handling risks of AI in practice. From the gathered perspectives, we derived 5 required characteristics for AI risk management methodologies (balance, extendability, representation, transparency and long-term orientation) and determined demands for clarification and action (e.g., for the definition of risk and accountabilities or standardization of risk governance and management) in the effort to move AI accountability from a conceptual stage to industry practice.

## 1. Introduction

The influence of Artificial Intelligence (AI) in our society is growing. Businesses are increasingly considering the use of AI models, as they offer new application possibilities. Therefore, simple approaches are being replaced by complex systems that can complement, or even surpass, human capabilities. This leads to a shift of company processes through AI systems that act independently and autonomously. However, these increases in both capability and complexity raise new questions as to how the predictions, decisions or actions of AI-based applications must be administered. Questions on who is to be held responsible for an AI system’s outcomes, thus, who is accountable for it, are frequently discussed. Particularly, as there are specific ambiguities and difficulties when attempting to hold someone accountable for an AI-based system’s results. Several responsibility gaps have been articulated for AI (Santoni de Sio and Mecacci, 2021). A culpability gap arises from the human desire to know the cause of an occurred harm and, especially if the cause is due to fault, to justify or punish it (Santoni de Sio and Mecacci, 2021). But there are limits to justification and punishment if the decision-maker is an automated system instead of a human-being. Further, the inability of ‘asking why’ that arises with certain AI systems creates a moral accountability gap, meaning that a system provider or operator can no longer be held morally responsible if they are unable to predict the behavior of a machine (Matthias, 2004; Santoni de Sio and Mecacci, 2021). This leads to the topic of accountability in AI still being highly contested and its way into practice unresolved.

Attempts are being made to deal with the problems of accountability through AI governance strategies (Raji et al., 2020). Particularly, since asking who to hold accountable primarily requires asking who to hold accountable for the harms caused by a system that works correctly or incorrectly (Wieringa, 2020), strategies regarding governing AI-related risks are broadly targeted. One example of risk-based governance is the recently proposed EU AI Act (Regulation, 2021/0106). It suggests a categorization of AI systems into risk levels based on their application field and foundation and imposes additional precautions to limit or adequately manage related risks. Similar concepts have been taken up and efforts made to further transfer them into practice. A large part of this contribution can be found among Standard Developing Organizations. The US National Institute of Standards and Technology (NIST) (2022, p. 1), for example, works on an AI Risk Management Framework, aiming at providing “a flexible, structured, and measurable process to address AI risks throughout the AI lifecycle”. In their Road Map Report on Artificial Intelligence, the CEN-CENELEC sets accountability among their priority standardization activities that are deemed “important to conduct soon” (CEN-CENELEC, 2020, p. 8). Risk management strategies are particularly emphasized as an important component thereof (CEN-CENELEC, 2020). However, it is also pointed out that risk governance for AI is not a concept that has to be rebuilt from scratch (e.g., National Institute of Standards and Technology (NIST), 2022). Approaches exist for other industries or branches and can be reconsidered or reused for AI (ISO 31000, 2020). For example, ISO 31000 generically introduces risk management processes as management tasks. IEEE/ISO/IEC 16085-2021 (2021) sets an international standard on risk management for system and software engineering along its life cycle processes. The essential elements thus seem to be understood and agreed and now need to be adapted to AI.

But precisely because fundamental concepts for risk management and accountability already exist, the question arises as to why there seem to be difficulties when transferring them to AI-based technologies. The above-mentioned responsibility gaps probably play a part in this, but what exactly are the hurdles to accountability and risk management of AI in practice and why do currently proposed risk management methods seem to not be suitable for practical application?

Our guiding research question in this endeavor is “How can (standard) risk management concepts be used to administer accountability in the context of AI?.” As we targeted practice-oriented research, we organized two exploratory and participatory workshops with experts and practitioners from the targeted fields, each workshop focusing on certain sub-aspects of our research aim. The purpose of Workshop 1 on “Accountability Requirements for AI Applications” was to further investigate risks and accountabilities arising from and with AI-based systems. We thus explored current challenges for AI accountability from a practitioner’s perspective and the ways in which risks are perceived in everyday work. Workshop 2 on “Risk Management and Responsibility Assessment for AI Systems” focused on AI risk governance, where we studied how existing AI risk management methods are applied in practice, what works well or not as well as which requirements for good AI risk management participants see from a practical perspective.

In summary, this work has three main contributions:

1. It gives an overview of the most pressing questions regarding accountability and risk governance issues as perceived in industry.
2. It provides insights into practitioners’ opinions regarding risk governance and brings their opinions at the center of the risk governance for accountability considerations.
3. It derives required characteristics for AI risk management methodologies as well as determines demands for clarification and actions to help move AI accountability from a conceptual stage to industry practice.

## 2. Accountability and risk governance in the context of AI

While accountability and risk governance have not (yet) been comprehensively clarified or adopted for AI-based applications in industry, their definition, elements and ways to practice have been examined within proposed concepts and frameworks. Let us therefore review what is needed to detail accountability as well as which aspects thereof currently proposed risk governance proposals can or cannot promise in the context of AI.

### 2.1. Accountability for AI technologies

Accountability has been broadly defined by Bovens (2007) as the relationship between an actor and the group (e.g., society) to which the actor holds an obligation to justify their conduct. It is what enables the evaluation of a stakeholder’s performance and pertains to their voluntary decision to provide information about their actions (Bovens et al., 2014). Wieringa (2020) has transferred this concept to algorithms, determining five key elements of algorithmic accountability: (1) the responsible actors, (2) the forum to whom the account is directed, (3) the relationship of accountability between stakeholders and the forum, (4) the criteria that are to be fulfilled to reach sufficient account, and (5) the consequences, good or bad, for the accountable parties. Two dimensions are therefore inherent to accountability, responsibility and the ability to provide justification or explanation for the measures taken to fulfill it.

Particularly the latter, justification, causes problems in the context of AI. During the creation, development and implementation of AI systems, different stakeholders need to be defined as accountable for possible formal or informal, good or bad consequences of the technology (Bovens, 2007; Olson et al., 2011). Explanations of the decisions made by the stakeholders, but also regarding the outputs of the AI system, are inevitable to ensure full accountability distribution (Wieringa, 2020). Explanations are therefore linked to the system’s ability to be transparent and explainable. However, the black box nature of complex AI systems is one major challenge (Bovens et al., 2014). The field of research surrounding explainable AI, or XAI, is growing to enable more transparent and thus more accountability-allowing systems (Gunning and Aha, 2019; Mittelstadt et al., 2019). Guidotti et al. (2018), for example, proposed four approaches to make an algorithm intelligible: explaining the model, explaining its outcome, inspecting the black box and creating a transparent box. While such efforts may provide a clear path toward transparency, they still cannot cover all the needs of transparency for accountability (yet).

One approach to overcome such issues is to detail decisions and actions for the different stakeholders (Henriksen et al., 2021). If the rational opinion prevails that an AI system cannot be responsible for its own actions, such as supported by the EU AI Act (Regulation, 2021/0106), the determination of a legal or physical person to be held accountable is inevitable. To determine such an accountable actor, certain processes must be made clearer, such as where the data feeding an algorithm comes from, how accountability can be ensured in case of decision-making mishap and, in case of unforeseen adverse consequences, in general (Fischer et al., 2020). The city of New York, for example, installed an Automated Decisions System Task Force to evaluate algorithms (New York City, 2018). The European Union’s proposed AI Act (Regulation, 2021/0106) provides guidance for obligations regarding the assessment and management of risks prior, during and after the implementation of a new system. Those initiatives show a great interest for the definition of accountable parties and actions to be responsible for. However, research deficiencies have been spotted when it comes to figuring out unclarities. While there are approaches to managing accountability in the context of AI, little attention has been drawn to standardized, global and sector-blind perspectives that are practical enough for industry demands (Tekathen and Dechow, 2013; Pollmann et al., 2014). Open questions arise also when it comes to the methods that enable the application of risk-aware accountability.

### 2.2. A risk governance approach to accountability

Risk governance is an activity “that requires consideration of legal, institutional, social and economic contexts in which a risk is evaluated, and involvement of the actors and stakeholders who represent them. Risk governance looks at the complex web of actors, rules, conventions, processes and mechanisms concerned with how relevant risk information is collected, analyzed and communicated, and how management decisions are taken” (Renn, 2008, cited after Stein and Wiedemann, 2016, p. 820). We can thus regard risk governance as realization of accountability for harms, as it includes considerations on all elements of Wieringa’s (2020) accountability definition. It is a sum of risk analysis and appropriate management (Renn, 2021) to establish strategies and actions for responding to unwanted events, their probability and consequences. Therefore, risk governance processes comprise pre-assessment, appraisal, characterization, evaluation, management and communication of risks (Renn, 2008), in the context of AI, risks that specifically arise from the characteristics of AI-based systems.

An impulse to take action toward risk governance can be imposed by policy. On an EU-level, regulations and directives have been put up to harmonize related activities. Legal frameworks, such as the General Product Safety Directive (GPSD) or Product Liability Directive (PLD) (Council Directive 85/374/EEC, 1985; Directive 2001/95/EC of the European Parliament and of the Council of 3 December, 2001). provide guidance and a cross-industry standard for organizations. They clarify obligations regarding risks, e.g., “economic operators shall place or make available on the Union market only safe products.” (Art. 5, GPSD), as well as specify their requirements, e.g., “‘safe product’ shall mean any product which, under normal or reasonably foreseeable conditions of use including duration and, where applicable, putting into service, installation and maintenance requirements, does not present any risk or only the minimum risks compatible with the product’s use, considered to be acceptable and consistent with a high level of protection for the safety and health of persons […]” (Art. 2(b), GSPD). Thus, national (or supranational) policies are a key component of organizational risk governance, as they provide sector-independent harmonization for risk assessment and characterization (Renn et al., 2011). However, scholars have pointed out limitations for their industry-wide fit when it comes to AI applications. For examples, as products within the scope of the PLD are defined as “all movables […], even though incorporated into another movable or into an immovable” (Art. 1, PLD), it has been discussed whether this applies for AI systems (Cabral, 2020). Further, it is argued whether compensation mechanisms of current liability regimes are ready for AI applications, as they limit claims to damage to the health or property of private users (Borges, 2021). These considerations emphasize that, while regulative approaches to risk governance are well-established, their applicability and extendability to AI-based systems needs to be clarified. Legislation has reacted with initiatives to modernize current frameworks to adapt them to the new circumstances (European Commission, 2022). In this endeavor, new regulations have been proposed that are specifically designed to address risks linked to AI-based systems, such as the previously mentioned AI Act, or even broken down to certain AI technologies, such as the regulations on “uniform procedures and technical specifications for the type-approval of the automated driving system (ADS) of fully automated vehicles” (Commission Implementing Regulation (EU), 2022/1426). While these initiatives reemphasize the need for updates and, when in force, hopefully can bring clarity on the fundamental concepts, in any case, their way into practice needs to be further detailed through materialization and operationalization.

Such realization is often guided by unified standards put up by authorized organizations and they are already widely acknowledged in the context of software development. Managing risks has been presented as a 4-step process entailing (1) Identification, (2) Access, (3) Control, and (4) Monitoring and Reporting (see, e.g., ISO 31000 standard; Jansen, 2018). Depending on the underlying intention, these steps can be implemented in different manners, e.g., ‘non-reactive’ approaches, where risks are mostly accepted, ‘reactive’ approaches, where risks are systematically addressed as they arise, and ‘proactive’ approaches, where risks are identified early and addressed before they show impact (Clarke, 2019). More specifically, in the context of software engineering, continuous risk management has been proposed as means to control resulting risks (Dorofee et al., 1996). “Continuous risk management is a software engineering practice with processes, methods, and tools for managing risks in a project. It provides a disciplined environment for proactive decision making to: continuously assess what can go wrong (risk); determine what risks are important to deal with; implement strategies to manage those risks” (Dorofee et al., 1996, p. 116). For example, Dhlamini et al. (2009) integrated risk management tools into software development projects and showed that these tools are necessary as project complexity increases. Risk management is used along with tools to keep risks within acceptable limits by identifying potential problems early, addressing them and eliminating them before they show effect (Wiegers, 1998). However, while established practices can limit risks of AI-software engineering, many AI-specific characteristics are overlooked. For example, deficits of providing reasoning for outputs that many AI systems have can impede effective risk management. Further, while ‘regular’ products are usually (or at least ideally) technically mature when placed on the market, self-learning systems evolve over time and require additional steering or monitoring. Traditional software risk control is not designed to ensure these measures.

To improve such issues, initiatives have been started to establish risk governance processes specifically for AI. Building off the definition of ethical principles, defining the ideal conduct an actor should have to strive for further than legal compliance (Floridi, 2018), several AI ethics guidelines have been proposed, such as the AI4People five principles for a good AI society (Floridi et al., 2018), the IEEE standards (Chatila and Havens, 2019), or the European High-Level Experts Group on AI’s work on trustworthy AI [High-Level Expert Group on Artificial Intelligence (AI HLEG), 2019]. With the increased research interest and debates, a current convergence toward five fundamental principles (transparency, justice and fairness, non-maleficence, responsibility and privacy) can be observed (Jobin et al., 2019). Therefore, current research efforts are directed towards how to operationalize these principles in practice (Morley et al., 2020). Governance frameworks specifically built around the risks of AI are therefore developed. Effectuation of the defined principles is ensured through risk assessments and management strategies. For example, checklists (e.g., AI HLEG, 2020; Algorithm Watch, 2021b), impact assessments (e.g., Ada Lovelace Institute, AI Now, and Open Government Partnership, 2021); or technical tools (e.g., Vakkuri et al., 2021; Felländer et al., 2022) are often proposed. They are encouraged to “build on existing policies and governance structures, use pragmatic and action-oriented terminology, focus on risk management in development and procurement, and empower employees through continuous education and change management” (Mökander et al., 2022).

This highlights that the need for new approaches has been widely acknowledged and efforts initiated. In fact, it has been pointed out that responsible development through ethical governance will be key to develop trust in AI systems (Winfield and Jirotka, 2018). However, on the other hand, while such processes exist in theory, they are just slowly finding their way to practice. The PwC 2022 AI Business Survey, for example, has found that “even though nearly every company has responsible AI ambitions, for each specific leading practice, fewer than half are planning action” (Greenstein and Rao, 2022). This further articulates the need to review current concepts of risk control to fulfill the new requirements of AI technologies. Certainly, there are already methods from other disciplines that can be reused for this purpose. This investigation of the transferability of methods from other areas to AI may help solve the currently perceived hurdles of determining accountability for AI-based systems. Indeed, the need for clear understanding of risks, followed by active response through risk management, seems to be one way to support the definition of responsible stakeholders and required actions. As risk-based approaches seem to be accepted and considered practical in industry, we argue that studying how risk governance concepts can be applied for accountability for AI can help overcome current challenges of impracticability.

## 3. Methodology

The aim of this research was to explore the use of risk governance to administer accountability for AI-based systems in practice, retrieving particularly which benefits and challenges currently arise with the application of AI risk governance. From the review of existing efforts, we see that the need for establishing accountabilities for AI-based systems has been clearly acknowledged, not only in theory. Concepts to operationalize it through risk governance, building off traditional techniques from software engineering, have been proposed. However, as we, at the same time, see difficulties of transferring and applying such approaches in practice, we argue that more evaluation of practitioners’ perspectives is required to ensure practicability of proposed concepts. We therefore address this demand with our findings of two workshops with AI practitioners on their views on accountabilities for AI-related risks as well as the currently proposed concepts to define and govern them.

**Figure 1:**
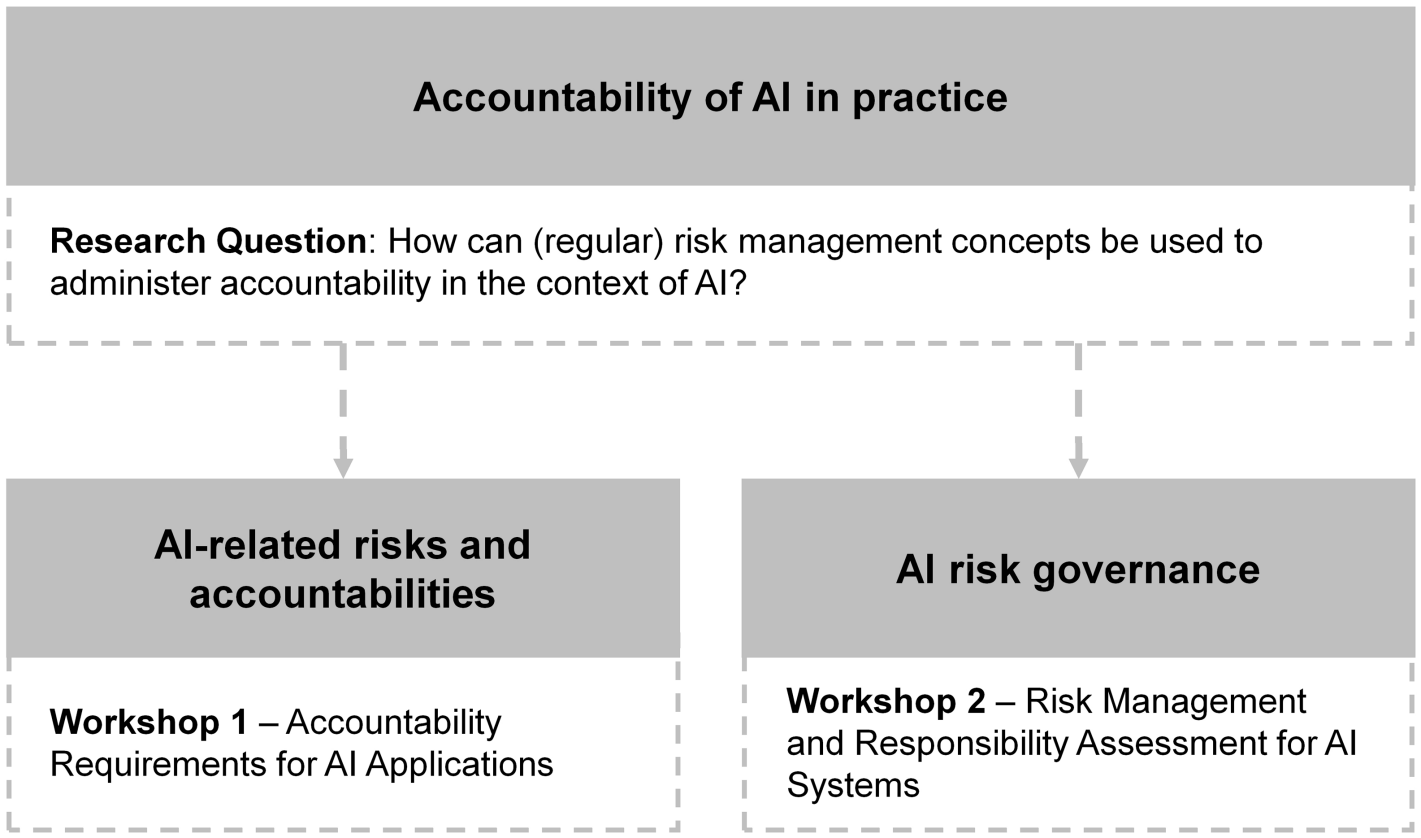
Overview of key considerations within and structure of this research.

More precisely, the purpose of Workshop 1 was to define and investigate the risks and accountabilities that arise with them. We looked at current challenges of risk responsibility attribution from practitioners’ perspective. In the second workshop, we studied current risk management methodologies, to what extent they are employed by practitioners, where they can help and what they lack from a practical perspective. Thus, discussions were turned toward risk governance investigating their use, strengths and limitations of risk management methodologies. Figure 1 outlines the research approach and key focuses of each workshop.

Our methodology was intended to be exploratory and participatory to reveal various sets of drawbacks for accountability and risk governance for AI. The choice of the workshop methodology is based on the participatory principle outlined by Vaughn and Jacquez (2020), allowing for meaningful engagement with target stakeholders and supporting the creation of relevant, meaningful research findings translatable into action.

### 3.1. Workshop 1: Accountability requirements for AI applications

**Figure 2:**
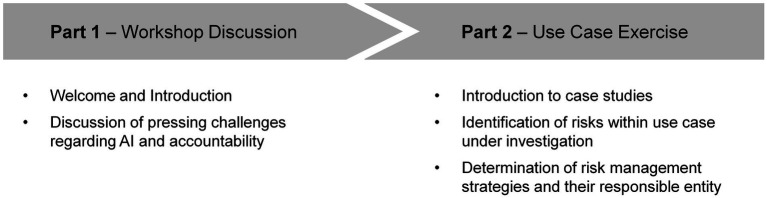
Agenda for the ‘Accountability Requirements for AI Applications’ workshop.

In the first workshop, we aimed at understanding the perspectives of practitioners from industry and academia on risks and accountabilities brought upon by AI systems with the aim to understand clearly the practitioners’ perspective when facing them, i.e., how do risks arise and which, their considerations of how risks can be appropriately addressed and, finally, who should be held accountable for such. The anticipated outcome was to build a clear, sector-blind map to then be able to identify appropriate risk management methodologies and responsible parties within or outside of an organization. This was targeted in two ways, first, directly exploring the challenges for accountability through workshop discussions with the participants from different backgrounds on their views on risks and, second, more practically, by studying concrete case studies to observe more clearly the different areas of concern in their opinions. Figure 2 shows the agenda of the workshop as presented to the participants.

#### 3.1.1. Participants

The group for the first workshop was composed of 18 participants, including three organizers. The participants came from different backgrounds: engineering, ethics, data science, policy, law and sustainability. Eight of them worked in academia, ten came from the private sector.

Participants were recruited through advertisement on professional social media platforms and personal network.

#### 3.1.2. Procedure

During Part 1, after a short introduction of the organizers and a brief presentation of the workshop’s background, fundamental definitions and motivation, the interactive workshop part started with an open, moderated discussion regarding the question ‘In your opinion, what are the most pressing challenges in the industry regarding risks of AI and accountability?’. Participants were invited to speak up or use the ‘raise your hand’-function of the online-meeting tool to join the discussion.

**Figure 3:**
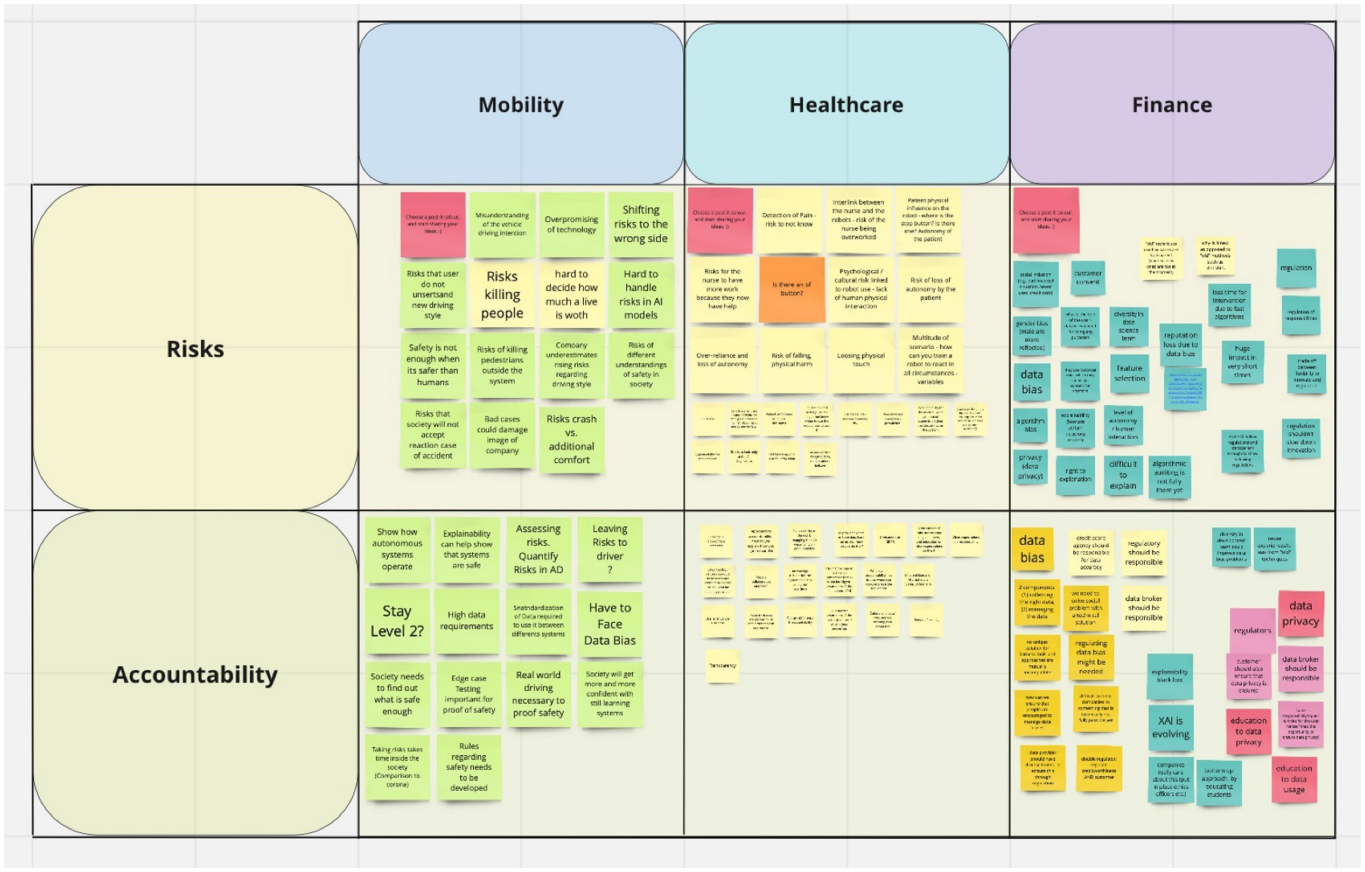
Screenshot of online collaborative “Miro-board” used for the use case analysis.

In Part 2 of the workshop, participants were presented three case studies (see Appendix A for details) on mobility, healthcare or finance topics. The mobility case study focused on the question of adjusting driving styles for autonomous cars, the healthcare case study questioned the use of robots to support elderly populations and the finance case study debated the algorithmic assessment of individuals’ creditworthiness. The use case analysis exercise was worked on in smaller teams in breakout rooms for which participants were asked to decide themselves which room, and thus use case, to join. The exercise consisted of two sub-tasks: (1) the identification of risks related to AI and ethics within the investigated use case, and (2) the determination of potential risk management strategies and their responsible entities. A “Miro-board”^1^ was used to record and structure the conversations and main outcomes of the exercises with the participants, illustrated in Figure 3. The “sticky notes” represent ideas and comments that were made during the exercise and were added by the participants or the organizers’ team member who attended and moderated the respective session.

### 3.2. Workshop 2: Risk management and responsibility assessment for AI systems

**Figure 4:**
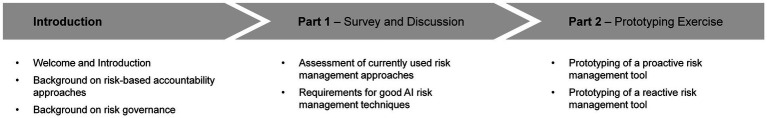
Agenda for the ‘Risk Management and Responsibility Assessment for AI Systems’ workshop.

For the second workshop, our aim was to understand in more detail the practitioners’ challenges regarding risk management tools for AI systems to clarify their pain points and gaps. Understanding with the first workshop their approach to accountability for risks, we now aimed at investigating the reason why the risks did not seem to be easy to mitigate with the available frameworks. Additionally, a major point of interest flagged early on in our research was the lack of clarity on who is accountable for what, which is why a second aspect of our workshop was to focus on responsibility attribution within and outside of the organization for each step of the risk management strategy implementation. Participants were thus first asked direct questions regarding current tools and their appreciation of those. Second, a prototype risk management process was developed in smaller groups to step by step investigate, for a chosen holistic AI-related risk, the required countermeasures as well as accountable stakeholders. Figure 4 shows the agenda of the workshop as presented to the participants.

#### 3.2.1. Participants

For this workshop, 19 participants were present, including three organizers. Except for the organizers, all participants worked in the industry. Their background covered the fields of law, AI ethics, sustainability and technical sciences. Some participants took part in both workshops, while others participated in only this one. The participation in both workshops was not required.

Participants were recruited through advertisement on specific social media platforms, contact of first workshop participants and personal network.

#### 3.2.2. Procedure

Participants were first introduced to the risk-based AI accountability approach. They were presented important regulations and policy papers published by the European Union, indicating which objectives and core values should be maintained and reached in AI applications, namely the High-Level Expert Group on Artificial Intelligence (AI HLEG) (2019) documentation on trustworthy AI and the AI Act (Regulation, 2021/0106).

During Part 1, participants were asked through ‘mentimeter’^2^, an interactive polls and word clouds tool, about their perception of AI-related risks in their daily practice as well as whether they use risk management tools or methodology to cope with those. Afterwards, participants were asked to give feedback in the form of multiple word clouds on their perceived limitations and benefits of existing risk management methodologies as well as challenges they face in managing risks today and what they would require in a good risk management tool, which were discussed afterwards.

**Figure 5:**
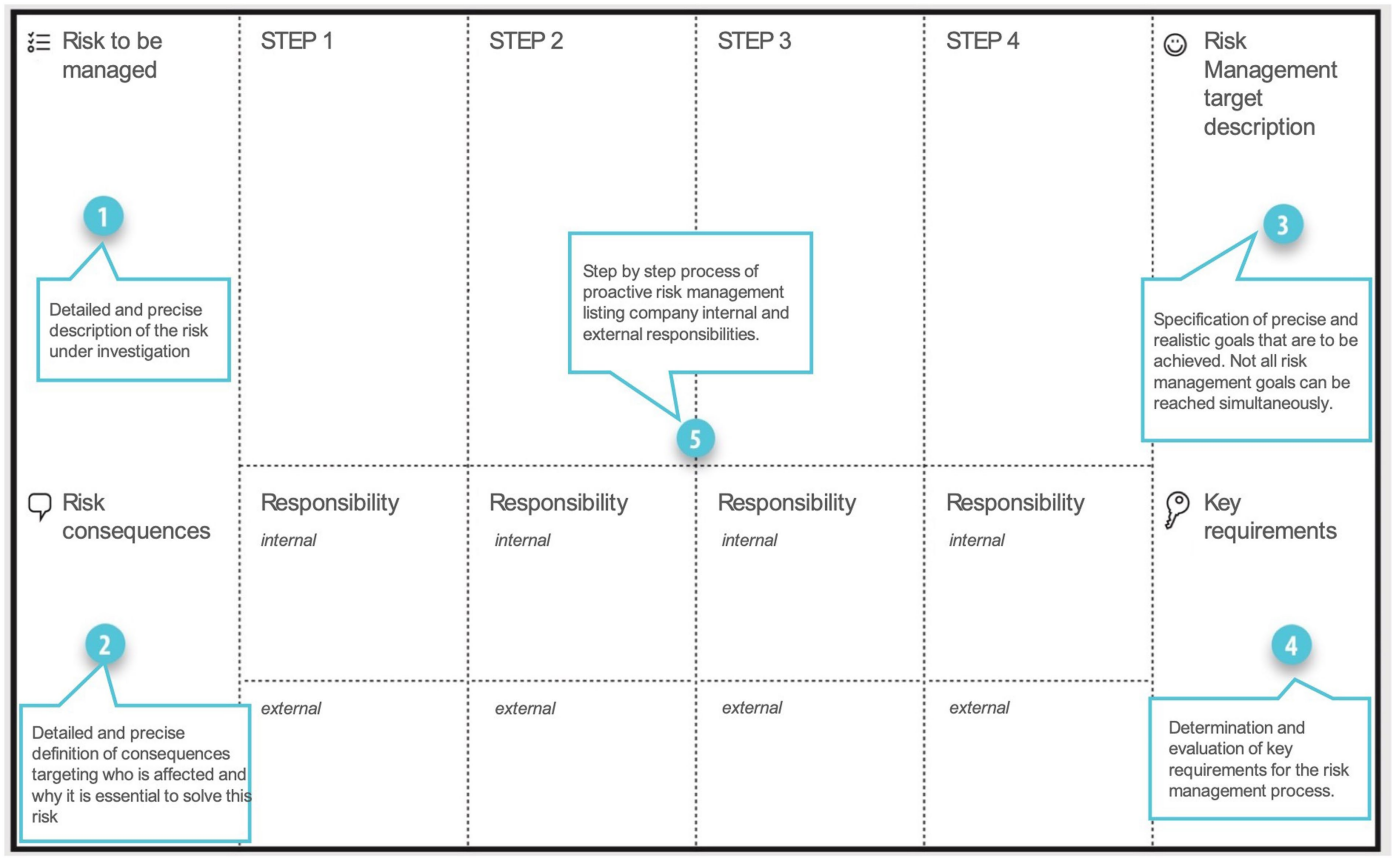
Outline for the AI risk management prototyping canvas.

Part 2 of the workshop dealt with the active prototyping of a risk management tool and took place in groups. Participants were introduced a risk management canvas as presented in Figure 5. The canvas was built off a common prototyping scheme as used in design practices^3^ and adapted to the workshop exercise. In break-out rooms, participants were asked to choose a risk that would be holistic to multiple AI-using sectors and to map the steps needed to manage the chosen risk within an institution. For each step, required activities as well as responsibility distribution they envisioned within and outside of the fictive organization were determined. Two scenarios were discussed by each group, a first one for proposing a proactive risk management methodology, in other words, the planned risk management activities to avoid or prevent harm from a risk in the first place (Clarke, 2019) and a second one building a reactive risk management methodology for the same risk, in other words, risk management activities to respond to or mitigate damages when a risk has created harm (Clarke, 2019).

### 3.3. Analysis

The findings were synthesized by going through the materials collected and notes from the discussions for both workshops. Additionally, for the first workshop, the Miro-Board content and, for the second workshop, the canvases content were integrated in the analysis. The authors discussed and structured the relevant outcomes. The main outcomes were identified through a ‘frequency of topics’ analysis and the review of arguments and details given by participants on their opinions during the discussions and exercises. The more the topics were mentioned by the participants, thus the more it was discussed, the more they were considered urgent and relevant. All topics mentioned, even if just once, were considered in the final outcomes. The proposed approach is similar to other articles building on workshop methodology in the area of ethics and technology (e.g., Danaher et al., 2017; Fosch-Villaronga et al., 2020).

## 4. Results and analysis

This section presents the results for the two workshop studies carried out. Filled material as well as discussion summaries will be presented and analyzed for each workshop to conclude findings resulting from the considerations. Their implications for theory and practice are compiled in the subsequent discussion section.

### 4.1. Workshop 1

In Workshop 1, risks perceived by participants in different scenarios and responsible entities for their mitigation were investigated. The main aim was to identify which risks are seen by practitioners in certain use cases as well as which patterns can be derived from this in order to retrieve further risks. Additionally, strategies for managing these risks were examined regarding options for specification and standardization as well as potential accountability determination mechanisms observed.

#### 4.1.1. Part 1: Workshop discussion

A summary of terms mentioned during the workshop discussion on pressing challenges regarding AI and accountability can be found in Table 1. Be reminded that the workshop was not technically recorded but notes were taken during the discussions to capture the participants’ opinions. Therefore, explanations presented in the second column of Table 1 are not direct quotes from the discussion but were paraphrased by the authors to explain the key points mentioned during the workshop.

**Table 1:** Summary of points mentioned by participants during the discussion on pressing challenges regarding AI and accountability.

Acceptance and Trust	Deployment and technology acceptance are two different things. Trust is key in acceptance; thus, we need to demonstrate trust.
Data Bias	AI systems must not be biased against certain groups in society. Non-discrimination and data quality needs to be ensured during development and deployment.
Education	Education is key. People need to be educated on the risks and safety of AI, data scientists and developers need to be educated on the ethical challenges of AI, and regulators need to be educated on current technological developments.
Explainability	There is a gap between what can be explained and what needs to be explained. Additionally, it needs to be ensured that people can understand what the system explains.
Implications	Accountability needs to be understood in terms of how but also which systems to design. Only because we can do something does not mean we should do it.
Privacy	How can high data privacy standards be fulfilled in AI systems?
Regulation	Detailed legal acts and legal cases are required.
Safety and risk	Technology can never be 100% safe. The question is, how much risk is bearable, what is safe enough and how can we determine suitable thresholds.

Evaluating and structuring the key arguments mentioned during the workshop discussion reveals that pressing challenges for AI accountability were mostly seen among two categories: (1) challenges w.r.t. the system design (data bias, explainability, privacy, safety, and risk) and (2) challenges w.r.t. the use of the system (acceptance and trust, education, implications, and regulation). Both dimensions thus seem to be important in determining accountability and should be considered independently (e.g., examine how biased data can affect responsibilities for the system’s outputs) as well as in relation to each other (e.g., how decisions regarding the system design influence accountabilities for the system’s use).

#### 4.1.2. Part 2: Use case exercise

In sub-task 1, the determination of risks related to AI and ethics, participants identified multiple, diverse risks within the different use cases. Appendix B shows the full results, presenting the identified risks as well as a short description w.r.t. the specific use case.

During the exercise we observed that participants predominantly focused on societal or end-user-related risks as well risks for organizations. Risks linked to the AI system itself, such as design-related decisions or the system’s accuracy, robustness and security, were less mentioned in the discussions. One reason for this could be a bias in the participants’ answers. Although great care was taken in the selection of participants to ensure a diversity of backgrounds (see Section 3.1.1), their previous experiences or the workshop context, which had an AI-ethics connection, may have led participants to adopt a more user-centered perspective in their risk considerations. Thus, our results do not imply that risks linked to the technology are less important but rather reconfirm the prevalence of perceived societal risks of AI systems.

**Figure 6:**
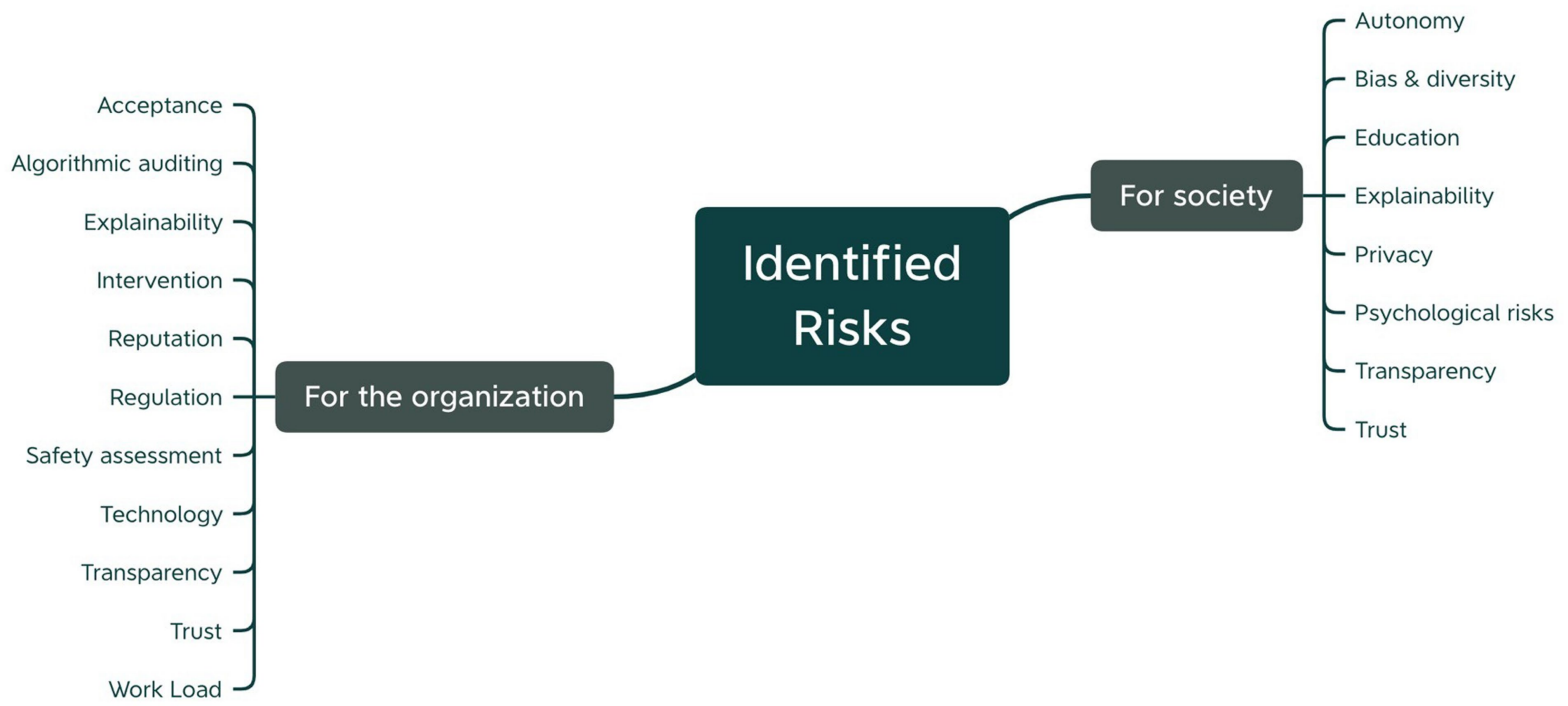
Risks identified by the participants during the workshop structured according to the actor they impact.

Based on the participants’ findings and our analysis, we propose to map AI-related risks along the two actors that are mainly impacted: the society (incl. End-users, other individuals and the general public) and organizations (incl. The AI provider, component producers and third-party organizations). Figure 6 demonstrates the resulting scheme for the risks identified by our participants during the workshop.

Appendix C gives an overview of results from the second sub-task of the use case analysis exercise, the definition of potential risk management approaches and the assignment of responsible actors for them. Due to time constraints, not all risks determined in sub-task 1 were discussed. Instead, certain risks were selected by the participants as examples.

**Figure 7:**
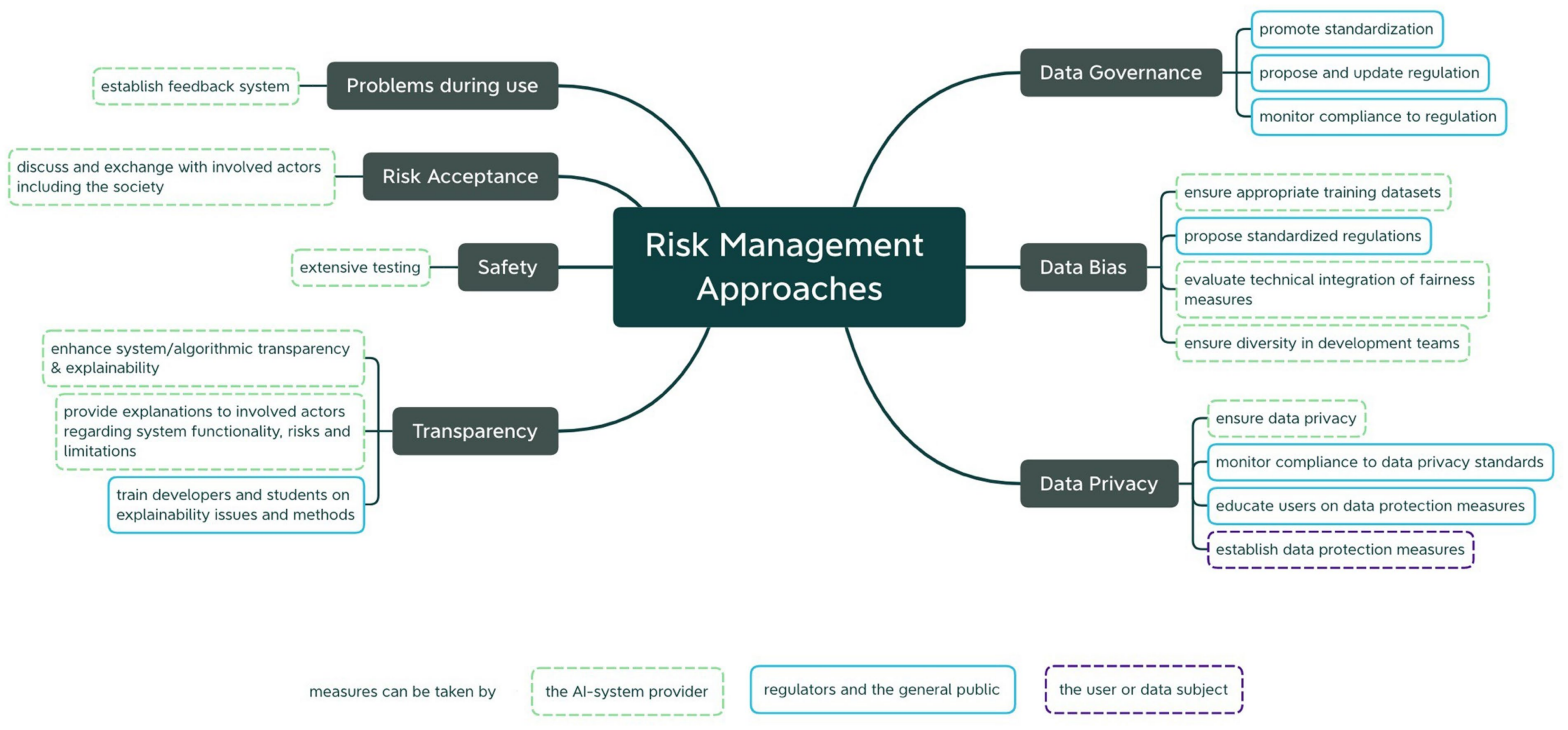
Summary of risk management approaches discussed during the use case analysis exercise for selected identified risks.

Analyzing the participants’ suggestions revealed a great variety of potential risk management measures. Figure 7 displays the investigated risks along with their respective potential risk management approaches that certain actors can take which have been discussed during the workshop. It demonstrates the great variety of risk management methodologies that participants have found during the exercise, ranging from technical methods, such as extensive testing or fairness-enhancing techniques, to non-technical measures, like inclusive debates with affected parties, detailed and understandable explanations or use manuals and promotion of team diversity. While most responsibilities were seen with the AI-system provider and regulators or general public, other stakeholders, like the user or data subjects, were found to be able to take action and aim at preventing or mitigating risks. In particular regarding data, participants proposed and wished for more regulative approaches to promote clarity and standardization. While the concrete design of certain methodologies was found to depend on the specific use case and context, an overall tendency, or at least the fact that certain measures are required, can be determined from a broader perspective, which is further reflected in Figure 7.

**Figure 8:**
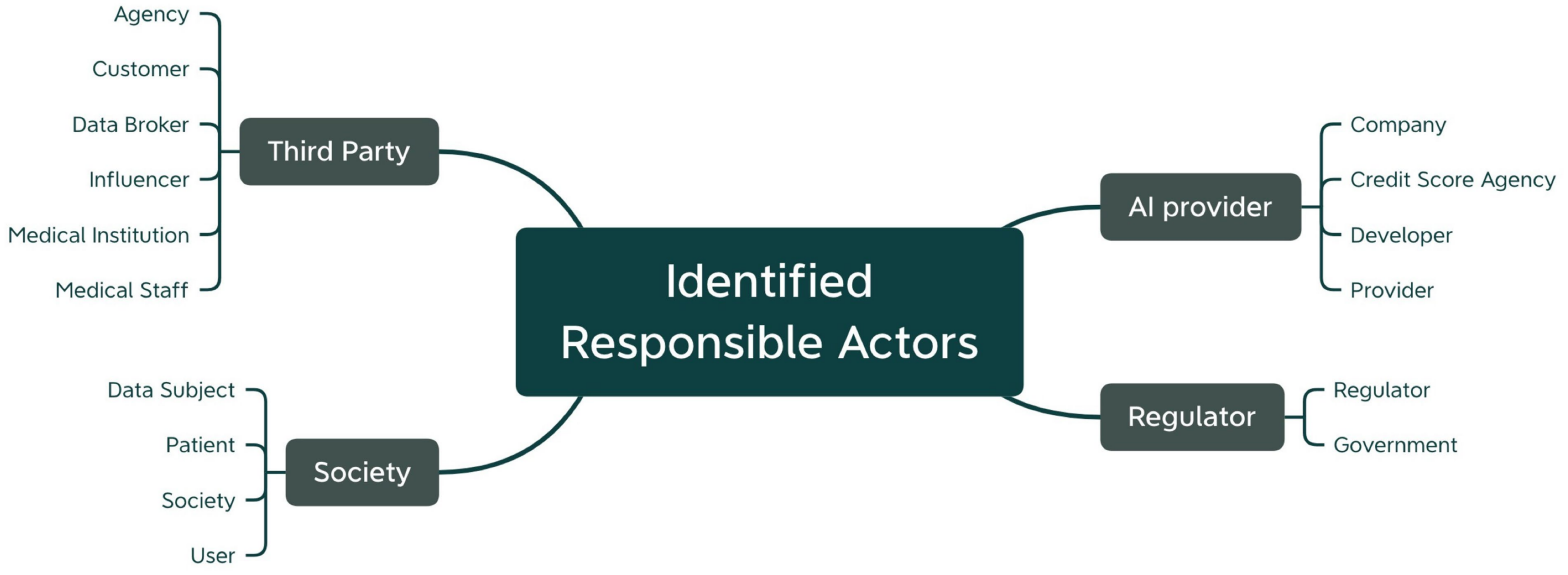
Responsibilities determined by the participants in the use case analysis exercise structured along the responsible actor.

The second point of interest in our analysis was how participants distributed responsibilities among the different actors involved. The participants’ answers revealed that they saw responsibilities for both, internal actors (i.e., the AI-system providing organization and its employees) as well as external stakeholders (e.g., regulators, the broader society or other third parties). Figure 8 provides an overview of which parties were pointed out by the participants during the use case analysis exercise.

As indicated before, AI-system providers were seen responsible for nearly all the investigated risks. Even though nearly all workshop participants had a practice-oriented background, it was not discussed further who within the company should be held responsible. Therefore, a precise responsibility-sharing scheme still needs to be detailed, which was one of our endeavors for Workshop 2. Regulators were mentioned second most often. While this reemphasizes the demand for more clarity regarding risk response approaches, it likewise indicates that there is still a gap of standardized, unified and at the same time sufficiently acknowledged frameworks for AI accountability. Finally, other actors were pointed out to bear some responsibility, among them third-party contributors and the broader society. Especially responsibilities for the user, data subjects or general public were frequently discussed during the exercise.

In summary these results thus indicate that the large network of stakeholders involved in AI development and use requires sharing responsibilities for risks fairly across various actors. Currently, this division is still unclear, and a uniform and standardized set of rules is desirable to offer more acknowledged guidance.

### 4.2. Workshop 2

The aim of Workshop 2 was to reveal insights on the daily practice of dealing with AI risks and risk management methodologies. The main interest was to investigate if and how participants perceived the risks of AI in their everyday work as well as how they cope with them. Ultimately this should lead to deriving requirements for good, useful and practical risk management approaches and the clarification of which challenges exist in their implementation.

#### 4.2.1. Part 1: Survey and discussion

The survey results obtained through online polls with the participants in the beginning of the workshop unveiled quantitative and qualitative insights on the participants’ perception of AI risks as well as practical application of risk management methodologies. They were followed by a mid-way and final discussion to receive more explanatory inputs from participants regarding their answers of the online poll. A summary report of the discussions regarding the participants’ opinions on AI risks and their perception during daily practice as well as requirements for good AI risk management can be found in Appendix D.

The two quantitative questions revealed that 13 out of 16 participants indicated to perceive the risks of AI in their everyday work (1 participant indicated ‘No’, 2 did not vote). To cope with these risks, 2 out of 16 participants reported using methodologies or technical tools (11 participants indicated ‘No’, 3 did not vote).

**Figure 9:**
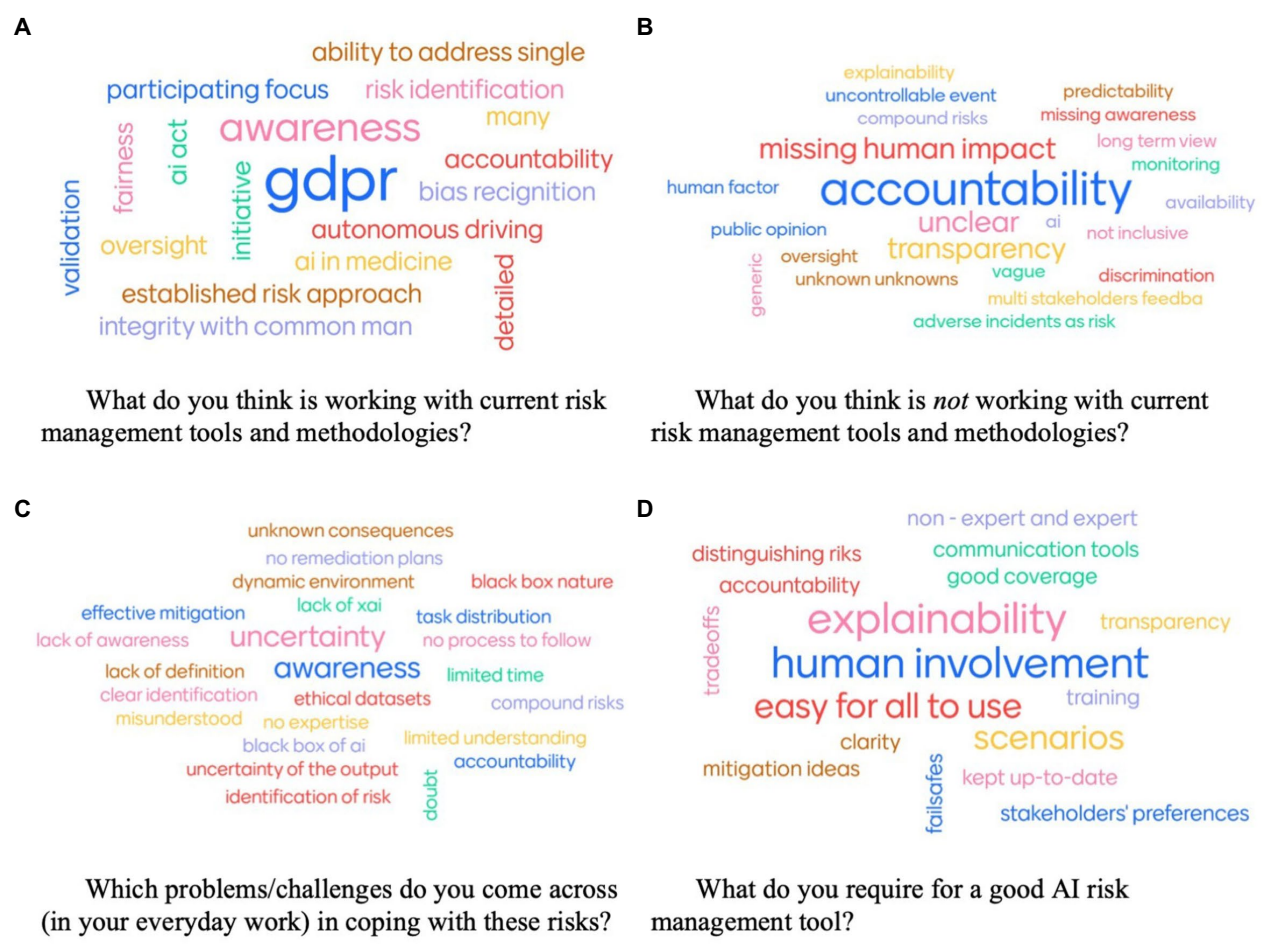
Word clouds of answers from participants to the four qualitative questions. **(A)** What do you think is working with current risk management tools and methodologies? **(B)** What do you think is *not* working with current risk management tools and methodologies? **(C)** Which problems/challenges do you come across (in your everyday work) in coping with these risks? **(D)** What do you require for a good AI risk management tool?

Further information on how to practically cope with risks linked to AI applications was found through qualitative questions. The word clouds to the four qualitative questions asked during the survey-part are displayed in Figure 9.

The participants’ quantitative responses show that there seems to be a need to manage risks linked to AI, however, current approaches are not considered suitable or satisfying enough to be applied in practice.

More concretely, certain aspects about risk management tools and methodologies have been pointed out as positive or ‘working well’ (Question A). GDPR explicitly has been highlighted as an effective tool (Regulation, 2016). Along with other mentioned terms, such as ‘detailed’, ‘oversight’ or ‘AI Act’, this emphasizes the participants’ demand for more standardized and recognized guidelines which seem to be considered powerful tools. Further, ‘awareness’ on the topic was mentioned as an important factor in the success of risk management. Presumably this accounts for both, awareness on risk management techniques, in addition to risks themselves, as sectors that are commonly considered riskier, like AI in autonomous driving or medicine, were particularly pointed out as being more effective w.r.t. risk management. Additionally, some available technical methods, such as tools for ‘fairness’ or ‘bias recognition’, were regarded useful.

Despite these positive aspects, several downsides have been mentioned regarding current risk management tools and methodologies (Question B). The most important issue seems to be a clear lack of accountability definition and distribution. Furthermore, the evaluation regarding the risks’ impact on humans does not seem sufficient in the current approaches, as they were considered to lack ‘multi-stakeholders’ feedback’, ‘public opinions’, ‘inclusiveness’, and ‘human factors’. Additionally, the lack of clarity (‘unclear’, ‘transparency’, and ‘explainability’) and the handling of unforeseen events (‘unknown unknowns’, ‘predictability’, and ‘uncontrollable events’) were criticized.

Regarding coping with risks in practice, participants were asked for challenges they perceive in their everyday work (Question C) as well as how these challenges could be resolved with risk management tools, thus, what they would require for a good AI risk management tool (Question D).

Three main challenges were mentioned by participants regarding practical handling of AI risks. Most importantly, they reported uncertainties regarding how to cope with perceived risks and responsibilities for it (‘uncertainty’, ‘no remediation plan’, ‘effective mitigation’, ‘task distribution’, ‘no process to follow’, ‘risk identification’, and ‘accountability’). During the discussion, this view was detailed, stating that risks are multidimensional and interrelated. Bias regarding how to approach risks, for example, due to a lack of team diversity, can further negatively affect the response processes. Second, a lack of awareness for risk coping approaches was mentioned as challenge (‘awareness’, ‘no expertise’, ‘limited understanding’, and ‘misunderstood’) and supplemented in the discussion with a misjudgment regarding urgency of many companies as well as a lack of sufficient resources to develop their own strategies and concepts, especially for smaller companies. Finally, a third key challenge mentioned relates to the black box nature of many AI systems. Intransparency of systems can particularly cause difficulties in managing risks, as sometimes risks are not identified correctly due to unintended consequences, and even if risks are properly determined, lack of understanding can lead to an inability to address risks appropriately (‘black box nature’, ‘black box of AI’, and ‘lack of XAI’).

Evaluating such challenges have led participants to specify certain requirements needed for good AI risk management. Specifically, a need for being understandable by all, experts and non-expert users, was determined (‘explainability’, ‘non-expert and expert’, ‘transparency’, ‘easy for all to use’, and ‘clarity’). Human involvement was further considered one key characteristic of such methods (‘human involvement’, ‘accountability’). A clear definition of risks with, for example, support from scenarios and use cases presenting mitigation ideas and failsafes in case of issues seems required to enable determining risk management processes as well evaluating trade-offs. Finally, tools should be adaptable and extendable, allowing for good coverage, stakeholder preferences and a long-term perspective. Particularly these characteristics were further detailed in the subsequent discussion. A call for clarity in coverage, i.e., which risks are employed by certain tools and how to treat the ‘unknown unknowns’ and standardization, i.e., resolve the currently scattered and incomplete nature of risk management tools, was expressed. Further the challenge of specification vs. generalization was discussed intensively and the unfavourability of a ‘one size fits all’ solution. Resolutions were proposed regarding balancing usefulness and detail with an extendable method, offering a generic model to avoid common mistakes and context-aware add-ons to be enacted for addressing specific issues being determined as most promising.

#### 4.2.2. Part 2: Prototyping exercise

**Figure 10:**
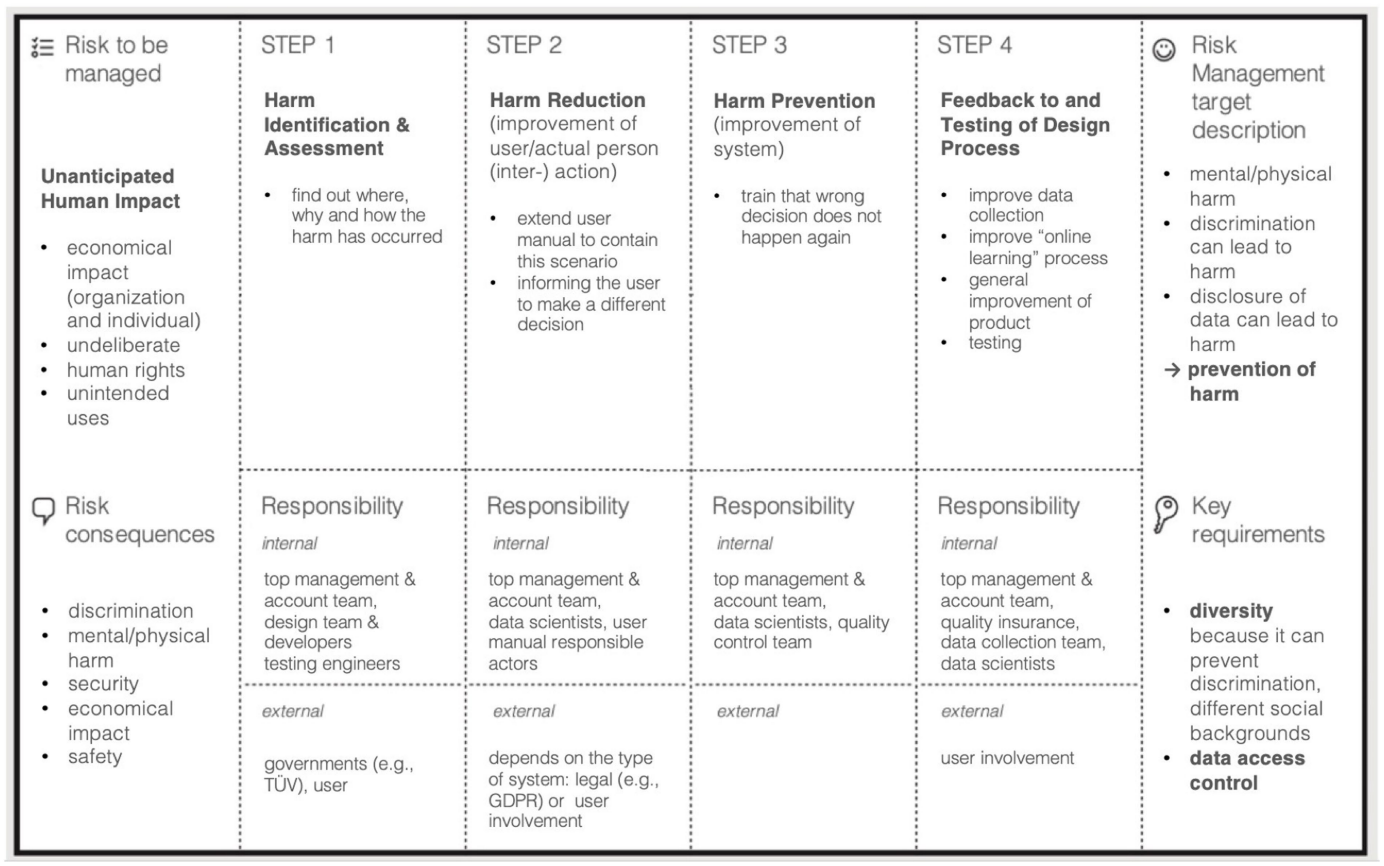
One of the prototyping canvases created during the workshop to exemplify the ideation process toward a reactive risk management technique.

During the interactive prototyping exercise, participants were divided into two subgroups and invited to brainstorm on the development of two risk management processes (reactive and proactive) for two different AI-related risks (fairness and unanticipated human impact). As an example, Figure 10 illustrates one resulting prototyping canvas which participants used during the exercise for structuring and noting down their ideas.

More specifically, Group 1 focused on fairness of AI-systems in different sector scenarios (health, education and emerging technologies) with the intention to mitigate discrimination caused by a lack of fairness for specific groups. For this, the target of the risk management process was determined as improved equity, inclusion and an increased consideration of the exact demographic of the target population in the system development. Group 2 aimed at managing unanticipated human impacts referring to unintended or undeliberate system use having negative effects on, e.g., human rights or economical aspects for individuals or other organizations. Consequences of unexpected impacts on humans were determined as discrimination, physical/mental harm or security and safety issues. Therefore, the targeted risk management aim was broadly defined as prevention of harm.

Developing the risk management processes, parallels can be found for both investigated scenarios as well as both examined management strategies. Essentially all sketched risk management processes followed four steps. First, a problem analysis is required involving a general problem definition, identification and assessment as well as conceptualization of processes and targets. Second, a reaction is planned, including strategies regarding problem improvement, harm reduction or implementation preparation, followed by the reaction execution referring to the actual evaluation and analysis of data or anticipated response implementation. The process ends with outcome testing, involving user tests and feedback loops. Application of these four steps slightly varied with the application to the use cases, however, a clear pattern was discernible.

**Figure 11:**
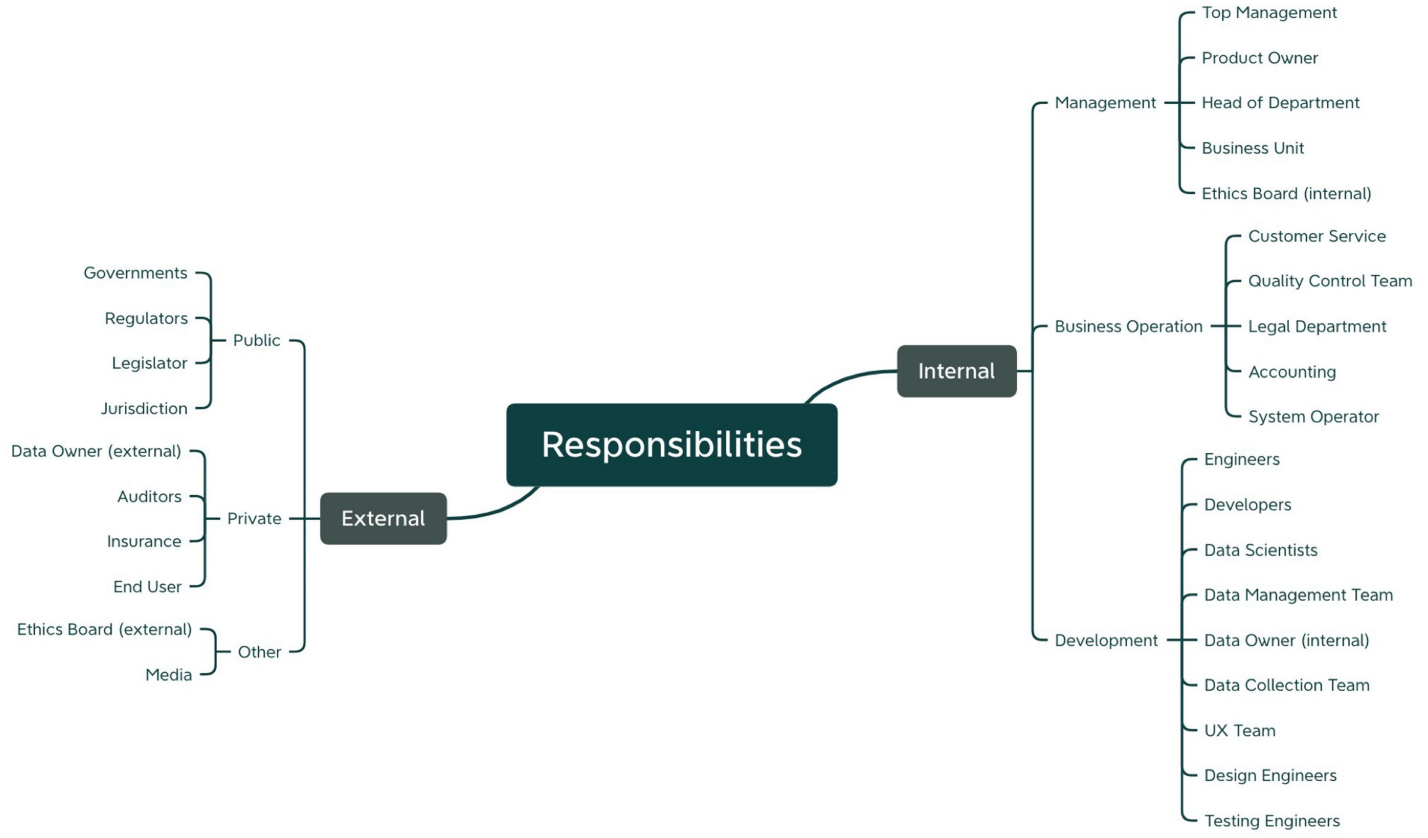
Summary of responsible actors determined for each step of the prototyped risk management process.

A final aim of the prototyping exercise was the definition of responsible stakeholders for each step of the sketched risk management process. Actors were asked to be determined within (internal) as well as outside (external) of the process implementing organization. Figure 11 provides an overview of all responsible parties mentioned among both subgroups in the exercise.

Internal responsibilities were mainly determined among the management, business operation and development. Responsible external actors were found in the public as well as private sector. Interestingly, in the two proactive approaches responsibilities for management were more found in the first and second step of the risk management process, dealing with the definition of goals and strategies, while the third and fourth step were predominantly seen as responsibility of development teams, implementation and testing of planned responses. This was perceived different with reactive approaches where responsibilities were shared across all teams throughout the whole risk management process, although the fundamental structure of steps was chosen similarly. Such peculiarities were not found for external actors, instead a need for action or monitoring was found at each step.

In summary, discussions during the prototyping exercise and shared participant opinions revealed valuable insights into practice-oriented risk management approaches. Established risk management ideas seem to be transferable to AI-related issues as well as suited for determining key risk management steps and responsibilities. A structured 4-step process was derived with responsibilities for internal and external stakeholders determined at each step.

## 5. Discussion

The two conducted workshops revealed various insightful conclusions from practitioners regarding how to connect standard risk management concepts to AI accountability. First, defining AI accountability comes with major challenges due to the way systems are designed and how they are used. When using risks as a heuristic to determine ‘what to be accountable for’, risks linked to AI systems can create implications along multiple dimensions mainly structured along implications for society and implications for organizations. Considering the use of risk management methodologies for administering AI accountability, there is a great variety in approaches and all affected actors can take action to prevent, manage and mitigate risks. Generally, established risk management ideas seem to be transferable to AI-related issues as well as suited for determining key risk management steps and responsibilities. However, although some techniques already work well, overall, recent AI risk management methodologies and tools are not suited for practical application due to their many downsides presented and therefore are hardly used in practitioners’ everyday work. Further, the concrete design of certain measures needs to be determined w.r.t. the use case and context, however, their general need, as well as broader implementation decisions can be determined generically. Finally, the large network of stakeholders involved in AI development and use requires sharing responsibilities for risks fairly across many actors. Currently, this division is still unclear, and a uniform and standardized set of rules is desirable to offer a more acknowledged guidance that is useful in practice.

### 5.1. Implications for theory

Our results reveal opinions from practitioners on how a connection between accountability and risk governance should be established and their evaluation regarding usability of currently proposed AI risk governance methods. These insights can be helpful for the development of new accountability frameworks, particularly those based on risk governance. Further, our findings can help overcome deficiencies of existing frameworks and guide their practical adaptation. In the following we therefore want to summarize how links between accountability and risk governance are currently seen in practice and which challenges arise with the practical use of current AI risk management tools. Finally, characteristics, content and methods for good AI risk governance are concluded to help adapt approaches to practitioners’ needs.

In the workshop discussions, the link between accountability and risk governance for AI has been identified within system design and system use of AI applications. Especially for challenges w.r.t. accountability for a system’s design, risk governance approaches seem to be a good response. In the prototyping exercise of Workshop 2, for example, accounting for data bias, or more generally fairness issues, has been investigated and a mock-up risk governance methodology to prevent related problems has been proposed (see also results for ‘unanticipated human impact’ in Figure 10). Proactive risk management strategies, like prevention, were found to be a suitable measure, a conclusion that seems further supported by the many efforts that have already been put up in this field by research or practice to advance their application, like XAI methods or data bias detection tools (Gunning and Aha, 2019). Based on such disaggregated, step-by-step considerations of risk management measures, responsibilities can be clarified. Implementation of prevention-measures for system design, however, benefits from the fact that system development, despite influenced by external factors, mainly lies in the area of competence of the AI-system provider. This might be more challenging when it comes to system use, as AI-system providers not necessarily have full power over how a user operates the system and its outputs. Nevertheless, risk governance approaches still seem promising. For example, considering the issue of missing education regarding AI use and its risks mentioned during the workshop discussion, use manuals or impact assessment summaries might be a reasonable solution. The link between accountability and risk governance becomes evident when revisiting our results from the use case exercise in Workshop 1, particularly those outlined in Figure 7, as accountabilities need to be considered not only for AI-related risks, but also for the management of those. When aiming to clarify accountabilities for AI, good risk governance approaches are thus inevitable. The question is therefore which disadvantages hinder their comprehensive application and how can they be overcome.

Summarizing the results from both workshops reveals challenges and drawbacks that our participants saw with current AI risk governance. First and foremost, the issue of defining accountabilities also impacts the creation and adoption of risk management measures, as unclarity regarding responsibility for a risk naturally leads to unclarity about responsibility for its mitigation. Second, the issue of transparency was mentioned in the context of two challenges. The black-box nature of AI systems was seen problematic to determine response mechanisms and the lack of understandability of risk governance measures in return hinders their effectiveness. Awareness for AI risks and management measures as well as the potential non-expertise of their operators can create issues. In addition, vagueness and unclear processes of risk governance approaches exacerbate such drawbacks. Finally, unanticipated consequences and unforeseen events have been mentioned as particularly hard to manage. Especially, if they entail negative impacts for humans, such risks should particularly be targeted.

Based on these identified drawbacks mentioned by practitioners during the workshops, we derive five key requirements that AI risk governance approaches should meet to be practically useful.

- *Balance*. One major issue mentioned was the lack of clearly defined processes that are highly adapted to the organization’s specific context and needs and thus would be easily implementable. However, at the same time the inability of many currently proposed AI risk management frameworks to be applicable in various scenarios and therefore the resulting unclarity about standardized and uniform procedures was pointed out as a particular downside. This argument is in line with findings from literature, as currently available frameworks in the EU context do not cover AI from a holistic perspective and cannot provide a sector blind understanding of arising risks [see, e.g., U.S. Government Accountability Office (GAO), 2021; Europol Innovation Lab and CENTRIC, 2022]. A key duty when designing risk management measures is thus finding a good balance between specialization and generalization. One suggestion for solving this quest is to develop a holistic fundament following standardized rules and, in addition, allowing for add-ons or extension to ensure adaptability per sector.
- *Extendability*. The second key requirement links to the dynamic nature of risks and the environment they need to be operated in. Risks or approaches to solve them can evolve over time, new risks can be revealed or arise in combination with newly developed technologies or regulations that need to be adhered to can emerge. Therefore, risk management recommendations should not be rigid, instead, they should be easily extendable and adaptable to new, perhaps even unforeseen aspects to stay practically useful also over time. This would also allow for a better identification and interpretation of the unknown knowns and unknown unknowns as presented by Bralver and Borge (2010).
- *Representation*. Risk management approaches should be holistic and comprehensive. Often a lack of completeness has been mentioned regarding risk identification and management. Many existing tools are highly specialized and can thus not guarantee an inclusive risk governance concept (e.g., Tekathen and Dechow, 2013; Pollmann et al., 2014). Especially, when it comes to negative impacts on humans, many risk management approaches do not seem to consider them appropriately. Input and feedback from different stakeholders, e.g., field experts or the global population, could promote the representativeness of risk governance approaches regarding various risks.
- *Transparency*. Transparency has been identified as a major condition to enable practical application of risk management approaches. The tools need to be understandable and usable by all, including expert and non-expert users. Further, transparency can help reveal responsibilities within the process and thus promote overall clarity regarding needed interventions. Therefore, developed methods should be as easily understandable as possible to foster their integration into existing structure and procedures.
- *Long-term orientation*. Finally, missing long-term orientation has been pointed out as deficiency of many existing methodologies. Continuous monitoring and updating, however, can benefit the overall countering of risks. Thereby it should allow for identification and prevention of unexpected or unintended risks, as risks can alter over time.

Besides the more generic characteristics that risk management methodologies should fulfill, certain concrete content and methods needed for effective risk management, and thus accountability definition, were demanded during the workshop. In line with the identified unclarity about accountabilities, recommendations on clear responsibility definition and distribution have been requested (see limitations in: Regulation, 2021/0106; U.S. Government Accountability Office (GAO), 2021; Europol Innovation Lab and CENTRIC, 2022; National Institute of Standards and Technology (NIST), 2022). This further emphasizes the need for precise standards that has been previously stated in literature or policy efforts (e.g., Algorithm Watch, 2021a; Circiumaru and Kind, 2022). Impact on humans (i.e., individuals, groups and society) has been mentioned to not be targeted enough, therefore a clear call for possibilities to examine and prevent human impacts has been raised. Further, communication tools for internal and external use were demanded. This could help raise awareness but also prove compliance with imposed obligations. Finally, more training opportunities, especially for unintended consequences and AI ethics in general were requested to allow for intervention regarding AI risks already early on.

Our findings re-emphasize that risk management concepts are generally a good measure for administering and fostering AI accountability as well as its current challenges for practice. Nevertheless, the identified drawbacks show that many of the already developed AI risk management approaches fail to meet all required features or clarification demands to be practically useful. Therefore, there is clearly a need to adapt risk management concepts to practical demands in order to strengthen their ability and usefulness for defining AI accountability and administering it in practice. In addition, risk-based accountability frameworks should account for these requirements and demands to be practically useful.

### 5.2. Implications for practice

Besides concrete requirements for risk management techniques, several ‘Calls for Action’ for regulators and organizations can be derived from the shortcomings of current risk governance that participants perceive in their everyday practice. Table 2 summarizes the 6 defined ‘Call for Action’-items.

**Table 2:** Summary of “Call for Action”-items for regulators and organization derived from the workshop discussions.

Provide clear definition of risks	Risks arising from and with the application of AI are manifold and multidimensional. A clear and standardized definition of ‘risks’ is inevitable to allow for effective risk governance.
Provide standardization regarding risk governance	Currently a large variety of risk governance approaches exists. Unification and standardization are important to give guidance on which risks to address and how.
Provide clear accountability frameworks	A lack of clear definition of responsibilities and accountabilities for risks and their management was expressed.
Generate transparent, widely understandable and practical methodologies	Several downsides were seen across currently proposed risk management methods. Particularly their transparent and practical application along the full process chain was regarded challenging.
Include human impact evaluations in risk management processes	Evaluation regarding impacts of risks for humans was expressed to be only partially included or insufficient. Increasing the importance of human impact analyzes throughout risk assessment and management techniques has been demanded.
Provide clarity about how to handle unforeseen events	Due to their unpredictability unforeseen events and risks arising with them are still challenging to solve in practice. Clarity regarding responsibility and how to deal with them is required.

Several of these items can or should be addressed through regulations, and therefore our findings are relevant for regulators and policy makers. Particularly, demands for standardization can most effectively be solved through policy efforts. Indeed, a reaction to some of the raised needs can already be found in the AI Act proposal (Regulation, 2021/0106). For example, clarity regarding risk definition has been proposed through the categorization of AI technologies into minimal, limited, high risk and prohibited practices. The concrete list of prohibited or high-risk systems along with the AI Act’s objectives specifying protectable principles and values gives insights on which risks must be addressed. In addition, the necessity of several of the identified calls for actions can also be seen in the AI Act. For example, Art. 17(1)(m) of the AI Act specifically obliges an accountability framework “setting out the responsibilities of the management and other staff with regard to all aspects listed in this paragraph” for high-risk AI systems. However, other than that, no specific standardized requirements for such accountability frameworks are stated. Further, the need for assessing impacts on humans is a core fundamental of the AI Act. A high-level of protection of fundamental rights and Union values is clearly stated within the draft’s objectives. This is reflected in the categorization of high-risk AI systems, which may be extended, among other preconditions, with systems posing risk of adverse impact on fundamental rights [Art. 7(1)(b), Regulation, 2021/0106]. In addition, Art. 29(6), for example, redraws attention to the obligation of high-risk AI system users to carry out a data protection impact assessment that the GDPR obliges (Regulation, 2016). In summary, the AI Act clearly articulates many of the above defined Calls for Actions and can therefore cover certain facets of standardization. However, some aspects are only vaguely addressed or left open for concrete adoption. While this promotes generalizability, it raises challenges regarding specific and standardized application. The aforementioned problem of a good balance of generalization and specification is thus not completely addressed, even with the proposed AI Act. Finally, it needs to be restated that the AI Act is still under development and not put into force. Therefore, concrete verifications regarding its practicability are yet to come.

Similar to challenges for regulators, a major take-away for the practical application of risk management methods in the industry seems to be a lack of transparency and clarity regarding risk governance. The need for clarification regarding the nature of risks seems to already have been heard and addressed by some organizations. Companies like BMW^4^ or Novartis^5^ have adopted principles or codes of conducts for the generation of trustworthy and responsible AI. While this can help to give a better and easier overview of relevant fundamental values, translating abstract principles to practical activities might still be challenging. Further, clarification regarding coping with identified risks has been particularly demanded (Chui et al., 2021). Standardized procedures can be imposed through, but also by the AI-developing organization itself, e.g., through establishment of clearly communicated accountability frameworks and development of reaction processes. Further, opportunities for education and training of responsible persons can help create awareness about risks and how to deal with them. Nonetheless, while some companies are already establishing or practicing such approaches, it seems that such processes are not yet fully developed and therefore practitioners still see a need for further action.

### 5.3. Limitations and future research

With this paper, our aim was to explore how risk governance methodologies can be used to administer accountability for AI systems. While the chosen workshop-based methodology allowed for exploratory conversations and unveiled also unexpected areas of discussions, certain limitations regarding methodology and overall research approach need to be noted.

A first limitation to be pointed out is the potential lack of depth on specific topics allowed through the workshop settings. Due to the exploratory angle of the research, this was not a problem for studying our research questions, however, to obtain detailed solutions for the identified issues, more in-depth investigations would be needed. A second argument for limitation could be the non-systematic procedure in the transcription and analysis of the conversations throughout both workshops. We thus do not qualify this work as deep-qualitative analysis but as an exploratory approach giving insights on the practical implementation. A final limitation of our study is the number of participants and their possible lack of representation of the practitioners’ population. With the aim to mitigate the impact of this last issue on the results, we made sure to have diversity in gender, sector and ethnicity of the participants.

Regarding future research, we noted that our workshop participants demanded more precise information on which risk governance methodologies to use when and how. We thus encourage researchers to dig dipper into the questions of risk governance and accountability distribution within each specific sector with a strong focus on understandability and usability of the tools proposed, as a need for more structured and explainable practices has clearly been identified. A qualitative literature-based comparison of tools available in multiple sectors, building off existing work such as Morley et al. (2020), to develop a “new and improved” version could help. More research could also be done on the realistic consequences of AI applications on populations to support the clarification of AI systems’ impacts on humans and society, as participants pointed out the lack of understanding in this area and the lack of implementation of such concerns in current frameworks. Finally, unforeseen events and unknown unknown risks will always be present when it comes to innovation. Therefore, strategies and approaches to uncover and handle them will be needed.

## 6. Conclusion and outlook

The aim of our research was to investigate how current risk governance approaches can contribute to solving the challenge of accountability in and for AI based systems. Risk-based frameworks seem to be common in addressing this issue and have been frequently suggested, the latest regulative effort being the proposed draft of the EU AI Act (Regulation, 2021/0106). However, given their currently rare adoption, existing risk governance frameworks seem to be unpractical in real industry scenarios. Therefore, the purpose of our work was to study current endeavors towards risk governance for AI accountability in practice. An exploratory workshop-based methodology was used to gather insights from practitioners on their organizations’ habits regarding handling risks of AI in practice. Both conducted workshops revealed a variety of perspectives and thus allowed deriving multiple findings regarding requirements of suited risk management methods as well as explanations or actions demanded by the practitioners to move forward. Particularly, we found that clarity and standardization were much desired. This is especially interesting because, despite some efforts from policy, research or industry have been proposed in this direction, such findings further highlight the need to complement rather high-level approaches with tangible methodologies. We suggest that more attention be paid to these aspects in future studies to finally move accountability for AI systems from a theoretical concept to actual practice.

## Data availability statement

The original contributions presented in the study are included in the article/Supplementary material, further inquiries can be directed to the corresponding author.

## Ethics statement

Ethical review and approval was not required for the study on human participants in accordance with the local legislation and institutional requirements. Written informed consent for participation was not required for this study in accordance with the national legislation and the institutional requirements.

## Author contributions

EH, AB, RT, and CL contributed to the conception and design of the overall research project and the conception and planning of the conducted workshops. EH, AB, and RT contributed to the preparation, realization, post-processing and analysis of the two workshops. Further, EH, AB, and RT contributed to writing, revising and approving the manuscript. All authors contributed to the article and approved the submitted version.

## Funding

This work was supported by Fujitsu Limited and the Technical University of Munich’s Institute for Ethics in Artificial Intelligence (IEAI). Fujitsu Limited had the following involvement with the study: determination of overall study goal and participation in the workshops. All authors declare no other competing interests.

## Conflict of interest

The authors declare that the research was conducted in the absence of any commercial or financial relationships that could be construed as a potential conflict of interest.

## Publisher’s note

All claims expressed in this article are solely those of the authors and do not necessarily represent those of their affiliated organizations, or those of the publisher, the editors and the reviewers. Any product that may be evaluated in this article, or claim that may be made by its manufacturer, is not guaranteed or endorsed by the publisher.

## Footnotes

^1^Miro Board used for the workshop: https://miro.com/app/board/uXjVOGvHe4s=/?share_link_id=373952986511

^2^ https://www.mentimeter.com/

^3^Building off: http://www.designabetterbusiness.tools/tools/prototype-canvas

^4^ https://www.bmwgroup.com/content/dam/grpw/websites/bmwgroup_com/downloads/ENG_PR_CodeOfEthicsForAI_Short.pdf

^5^ https://www.novartis.com/about/strategy/data-and-digital/artificial-intelligence/our-commitment-ethical-and-responsible-use-ai
