# Supplementary material for: Investigating accountability for Artificial Intelligence through risk governance: A workshop-based exploratory study
Source: Front Psychol. 2023 Jan 25;14:1073686. doi: 10.3389/fpsyg.2023.1073686 (PMC9905430; doi:10.3389/fpsyg.2023.1073686)
Supplement: Supplementary file 1 [file Data_Sheet_1.DOCX]

Supplementary Material

# Appendix A – Use cases

| **Topic** | **Use Case Description** |
| --- | --- |
| **Mobility** | A vehicle manufacturer has conducted a study on satisfaction with its own self-driving vehicles. The study found that the biggest negative point was the vehicle's slow driving style. Due to their impatience, some customers prefer to drive independently and faster than autonomously. The vehicle manufacturer considers matching the risk through driving characteristics to that of a human driver in situations where there is always some risk. |
| **Healthcare** | An elderly care home decides to use healthcare robots to support the nursing staff in their daily tasks. The robot can help lift and help individuals walk from point A to point B under a nurse’s supervision. It adapts its posture to the weight and height of the patient. |
| **Finance** | A credit score agency developed a new and unconventional AI-enabled algorithm that, based on personal features, assesses the creditworthiness of people and offers this information to other companies, such as from the finance and insurance industry. The algorithmic model is neither disclosed to customers that buy the credit information nor to the public that is subject to creditworthiness investigations. |

# Appendix B – Risks identified by the participants during the use case analysis exercise for the three discussed use cases.

| **Use case: Mobility** | |  |
| --- | --- | --- |
| **Acceptance** | Changing the driving style and the risk of the overall technology could lead to a debate how much a human life is worth. The debate could slow down the overall technology development. |  |
| **Explainability** | Today AI algorithms are opaque and therefore carry risks. The user and the developer need to know what the system is doing in specific situations to debug the system or to react to misbehavior. |  |
| **Regulation** | The company must ensure that the applicable laws continue to be complied with the adaptation of the driving style. |  |
| **Reputation** | The new application could lead to additional deaths in the traffic. Even if the technology is considered safe, society may not accept the damage. Subjective opinions could harm the image of the entire industry. |  |
| **Safety Assessment** | Changing the system properties poses a strong risk. Every new feature needs to be tested and validated to ensure that the system works as intended. Changing the driving style could shift the overall risk e.g. to pedestrians. |  |
| **Transparency** | Customers and society could have a different understanding of safety and how safe a system has to be. Intransparency of the system could lead to further risks. |  |
| **Trust** | The vehicle's new AI enabled driving style could confuse passengers and other traffic participants. If the driving style is not intuitive, new problems regarding trust could arise. |  |
| **Use case: Healthcare** | |  |
| **Autonomy** | The patient could be at risk to see the robot’s use imposed on them without enlightened consent, or in contrast be requiring the robot when unnecessary, and thus not recover or loose movement abilities prematurely. Additionally, the autonomy of the nurse to decide to use this new tool or not has been identified as a risk. |  |
| **Education** | As mentioned earlier, there is a risk for nurses not to know the tool as they would like, creating situations of distress for them. This also applies to patients who will be using the robot, which are at risk of not fully understanding the extend of its usefulness, its workings, and which medical data are necessary to its functioning. Additionally, because the robot would require supervision from the nursing team, risks lie with nurses’ “undereducation” on the robot technology. |  |
| **Privacy** | As for any AI-powered technology, patient’s data will be required to have the robot run as it should. Due to cybersecurity and simple use risks, data privacy to external entities, and non-medical staff internal entities are to be considered. |  |
| **Psychological** | Due to the use of a robot for movements, the reduction of human physical interaction might be a wellness risk for the patients. |  |
| **Technical** | The robot could present a risk to the user’s physical well-being if it was for example not equipped to recognize signs of physical pain, or trained on data sets showing bias, thus having the robot not able to properly assess variations in weight and height of a person due to individual characteristics. |  |
| **Workload** | Management could believe the nurses can now take more work on as they receive technological help, putting the nurses at risk of overwork. |  |
| **Use case: Finance** | |  |
| **Algorithmic auditing** | Automated auditing is highly encouraged for such a use case, given its operation in the finance sector. However, methods for algorithmic auditing are still to be advanced. |  |
| **Autonomy** | The algorithm’s level of decision autonomy and degree of human interaction or intervention can pose risks, in particular given the fast operation times. |  |
| **Bias & Diversity** | Several forms of bias (e.g., gender/socio-economic bias in data/algorithm) can impact the algorithm’s fairness and effectiveness and, hence, lead to reputation loss of the engaged actors. |  |
| **Intervention** | The algorithms efficiency and speed leads to less time for intervention and therefore large impacts in short times. |  |
| **Explainability** | Missing transparency can cause problems, in particular, for operation in the finance sector. Transparency and explainability are needed to ensure and demonstrate compliance with regulations |  |
| **Privacy** | Data privacy poses a strong risk. Consumer consent can be challenging, in particular, if the algorithm uses historic data. |  |
| **Regulation** | Regulations are needed to define responsibilities, however, there is a trade off between flexibility/speed of innovation and innovation governance. |  |

# Appendix C – Risk management strategies and responsibility distribution identified by the participants during the use case analysis exercise for the three discussed use cases.

| **Use case: Mobility** | |  |  |
| --- | --- | --- | --- |
| **Data Governance** | A *regulation*of data standardization might be needed. The quantity and quality of data are critical to the success of AI systems. In order to share data between different companies and applications, *global regulation* and *standardization* is needed. |  | *Responsible actors:*companies, agency, regulator, data broker |
| **Risk Acceptance** | The society needs to *discuss edge-case scenarios* and how safe an AI system needs to be. A new human driver for example also causes higher risks than an advanced driver. How much risk is acceptable and is the society willing to accept systems that are still in a learning process? |  | *Responsible actors:*society, regulator, government, influencer |
| **Transparency & Safety** | The stakeholders need to understand how the system works and handles decisions. To increase the trust and acceptance of the customers and regulators the AI *application needs to be* *transparent*. Nevertheless, the system must be *tested extensively* in the real world to be considered safe and reliable. |  | *Responsible actors:*company, regulator |
| **Use case: Healthcare** | |  |  |
| **Data Governance** | The provider should ensure that *appropriate training dataset* are used depending on the target populations for a most adequate performance and fairness demonstration. Moreover, regulators are to *monitor the compliance*to current GDPR regulation and reflect on the possible need to make it *evolve* to fit best with AI technology evolution. |  | *Responsible actors:*regulator, provider |
| **Problems during use** | Ensuring a proper *feedback system*between the AI, the provider, the developer, and the users is necessary to fix issues when they arise and meliorate the product to the specific needs of the target population, taking into account multidisciplinary point of views, cultural aspects, and full transparency on all sides. |  | *Responsible actors:*developer, provider, users |
| **Transparency** | The providers should provide clear and detailed *explanations* *of the limitations and risks*, hardware, and software workings of the tool to all actors involved with it, whether the patients, the medical staff, or the institution buying it. Additionally, situations in which the end-user or supervisor are to be accountable for the tool need to be defined according to the law and specific regulations by the provider. |  | *Responsible actors:*developer, provider, patients, medical staff, medical institution |
| **Use case: Finance** | |  |  |
| **Data bias** | A *regulation of data bias* might be needed. However, challenges arise with this approach, as *technical integration of fairness in data and algorithms* is still an ongoing field of research. *Diversity in development teams* could raise the awareness for data bias issues and, hence, help managing this risk. |  | *Responsible actors:*credit score agency, regulator, data broker |
| **Data privacy** | The actors developing, deploying and using the investigated algorithm should ensure that *data privacy is provided*. Regulators should check *compliance to data privacy standards*. The data subjects simultaneously bear some responsibility for enabling all possible measures to *protect their own data*. Therefore, education to data privacy and data usage is key. |  | *Responsible actors:*credit score agency, regulator, data broker, customer, data subject |
| **Explainability** | XAI techniques are emerging and companies are increasingly interested in fostering *algorithmic transparency*. *Education* can help strengthen and accelerate awareness and mitigation of explainability issues. A “bottom-up” approach to *train students on explainability issues and methods* could help solving this issue for the future. |  | *** |

* responsible actors have not been determined during the workshop due to time constraints

# Appendix D – Summary of points mentioned by participants during the discussion on how to cope with AI risks in practice.

| **AI risks and how to cope with them during daily practice** | |  |
| --- | --- | --- |
| **Bias** | Bias is a very important issue and risk. External opinions or expert advisors might help address and avoid them. |  |
| **Diversity** | In order to adequately address risks of AI applications, various stakeholder perspectives must be sufficiently involved. Therefore, a diverse team from different cultures and disciplines is preferable. |  |
| **Focus on People** | Handling AI risks must serve people, not things. The focus should be on the interests of people and society, also considering ‘the bigger picture’, in order to avert harm. |  |
| **Multidimensionality** | There is a multitude of risks that come with AI applications and all of them impact humans. We should look at all the risks in total and consider them altogether to be able to grasp their impacts. |  |
| **Unintended Consequences** | It is not easy but at the same time important to know what else the system could be used for. Investigating one's own product regarding its deficiencies and in terms of unintended use, e.g., through workshops, is needed. |  |
| **Urgency & Accessibility** | Many companies do not perceive the urgency of coping with AI risks. In addition, smaller companies often do not have sufficient resources to develop their own strategies and concepts. |  |
| **Requirements for good AI risk management** | |  |
| **Coverage** | It needs to be identified if all possible risks are covered. The ‘unknown unknown’ is a big issue for accountability. |  |
| **Education & Explainability** | Explainability of AI products is one of the most important points regarding accountability. To achieve this, stakeholders need to be educated on how to use and supervise AI applications. |  |
| **Extendibility** | New fields always have new requirements and demand adaptions or changes. There will be new risks in the future, so methods cannot be static but need to be adaptable to upcoming aspects. |  |
| **‘One size fits all’** | A ‘one size fits all’ approach is not desirable, and hardly achievable, for AI risk management. A generic model to avoid common mistakes and context-aware add-ons to be enacted for addressing specific issues seems more practical. |  |
| **Specification vs. Generalization** | You can’t be generic and specific at the same time, as different systems have different characteristics or features, e.g., different sectors have different risks. Balancing usefulness and detail is therefore very important, although it might be difficult to reach. |  |
| **Standardization** | AI ethic assessments are scattered and methods are not complete. Currently there is no standardization. Therefore, the development of more comprehensive, end-to-end methodologies should be the focus of the next years. |  |
